# PLA/PA Bio-Blends: Induced Morphology by Extrusion

**DOI:** 10.3390/polym12010010

**Published:** 2019-12-19

**Authors:** Violeta García-Masabet, Orlando Santana Pérez, Jonathan Cailloux, Tobias Abt, Miguel Sánchez-Soto, Félix Carrasco, María Lluïsa Maspoch

**Affiliations:** 1 Centre Català del Plàstic (CCP)-Universitat Politécnica de Catalunya Barcelona Tech (UPC-EEBE), C/Colom 114, 08222 Terrassa, Spain; violeta.garcia.masabet@upc.edu (V.G.-M.); jonathan.cailloux@upc.edu (J.C.); ccp.tobias.abt@gmail.com (T.A.); m.sanchez-soto@upc.edu (M.S.-S.); maria.lluisa.maspoch@upc.edu (M.L.M.); 2 Department of Chemical Engineering, Universitat de Girona (UdG), Campus Montilivi s/n, 17071 Girona, Spain; felix.carrasco@udg.edu

**Keywords:** PLA, BioPA10.10, PLA/PA Bio-blends, in situ MFCs

## Abstract

The effect of processing conditions on the final morphology of Poly(Lactic Acid) (PLA) with bio-based Polyamide 10.10 (PA) 70/30 blends is analyzed in this paper. Two types of PLA were used: Commercial (neat PLA) and a rheologically modified PLA (PLA_REx_), with higher melt elasticity produced by reactive extrusion. To evaluate the ability of in situ micro-fibrillation (μf) of PA phase during blend compounding by twin-screw extrusion, two processing parameters were varied: (i) Screw speed rotation (rpm); and (ii) take-up velocity, to induce a hot stretching with different Draw Ratios (DR). The potential ability of PA-μf in both bio-blends was evaluated by the viscosity (*p*) and elasticity (*k’*) ratios determined from the rheological tests of pristine polymers. When PLA_REx_ was used, the requirements for PA-μf was fulfilled in the shear rate range observed at the extrusion die. Scanning electron microscopy (SEM) observations revealed that, unlike neat PLA, PLA_REx_ promoted PA-μf without hot stretching and the aspect ratio increased as DR increased. For neat PLA-based blends, PA-μf was promoted during the hot stretching stage. DMTA analysis revealed that the use of PLA_REx_ PLA_REx_ resulted in a better mechanical performance in the rubbery region (*T* > *Tg*_PLA-phase_) due to the PA-μf morphology obtained.

## 1. Introduction

The generation of polymers derived from renewable sources, also called bio-based polymers, has been an important field of research due to the role that these ecofriendly polymers play in the reduction of plastic residues as a source of pollution, as well as the reduction of carbon dioxide production, which leads to a decrease in the carbon footprint of its lifecycle [1,2]. Over the past decade, bio-based polymers, such as Poly (Lactic Acid) (PLA), have gained interest as a substitute for conventional fossil-based polymers in biomedical and commodity applications. Its main features are its biodegradable nature, the decrease in the CO_2_ footprint associated with the product, and the non-toxic residues released during processing [3,4,5,6].

Despite its great potential, PLA still has limitations, such as brittleness, slow crystallization kinetics, reduced service temperature range, high instability during processing where good melt strength is required, among others. There is a large amount of research dedicated solving these drawbacks in order to expand the application window and become a commodity or even engineering thermoplastic [7,8,9,10,11]. 

Among the strategies considered, melt blending of PLA with other polymers has been a practical and economic way for improving the properties of PLA [6,8,10,12,13]. Recently, blends with bio-sourced polyamides (PAs) have gained interest, regardless of their limited miscibility [14,15,16,17,18]. The control of the resulting morphology of these blends has proven to tailor their properties, such as mechanical, crystallization kinetics, and processability.

It has been demonstrated that, for immiscible blends, a fibril morphology of the dispersed phase, suitably compatible and oriented during processing, could generate a reinforcing effect. This type of induced morphology is referred to as in situ microfibrillated composites (MFCs). Some successful examples of these systems are blends using polyethylene terephthalate (PET) acting as the dispersed phase in fibrillar form [19,20,21], and recently PLA/PA blends [14,16,22]. In all of these cases, an increase in properties, such as fracture toughness, particularly during crack propagation, melt strength, and even enhancing the crystallization of the matrix has been reported [20,21,23,24]. 

A common technique to produce these MFCs is based on preparing a polymer blend of the matrix A and the dispersed phase B. Then, the blend is drawn, either in melted state (hot drawing) or already solidified (cold drawing), which transforms the spherical domains of B into highly oriented micro-fibrils with an increased slenderness. The blend can be used in pellet form and produce isotropic parts, fulfilling the following thermal processing condition: *T*_m_(A) < *T*_proc_ < *T*_m_(B).

At this stage, the matrix melts while the microfibrillar dispersed phase maintains its shape. Subsequently, these pellets are used in the final forming process (part production), thereby obtaining a product with enhanced mechanical performance. The success of this process is governed by the composition, rheological behavior and compatibility of the used polymers [20,21,25,26].

Until now, few studies have been developed from a rheological point of view. Some of the works worth mentioning are Ding et al. [27] with PLA/PCL blends and Yousfi et al. [16] with PLA/PAs blends. Both studies confirm that through the appropriate choice of blend components in terms of viscosity and elasticity ratio, flow conditions, and interfacial tensions, the production of in situ fibrillation morphology allows an improvement in the thermomechanical performance at a reasonable cost without sacrificing weight and ability to be recycled. Thus, a rheological analysis of parameters, such as viscosity ratio (*p*) and elasticity ratio (*k’*) could be a way to estimate a morphology based on the behavior of the pristine polymers, even to try to control the final behavior obtained to produce a desired morphology like MFC’s [16,28].

Fused Deposition Modeling (FDM) can take advantage of the MFC technique. The produced parts suffer from low mechanical properties and low surface quality, compared to injection molded parts. The mechanical properties and the surface roughness of parts manufactured by FDM are controlled by the adhesion quality of filaments and the global porosity of the piece obtained. They both mainly stem from the flowability and surface tension of the polymer. A controlled generation of a fibrillar morphology based on knowledge of the rheological behavior of the materials involved in the blend at the processing conditions used can contribute to a substantial improvement of the piece as far as mechanical behavior is concerned [22,29].

To comprehend the parameters affecting the morphology generated in blends requires a deep understanding of the physical process during the mixing condition by which the melted blend is exposed. So, it is useful to use the microrheology as a guide. Many authors have studied the fluid mechanics of the mixing process of two immiscible fluids, evaluating the effect of the different types of uniform flow fields applied (shear or elongational) in the matrix and the dispersed phase in form of droplets [28,30]. During the flow of the system, the change in geometry of the dispersed phase will depend on the ratio between the hydrodynamic forces dictated by the viscosity of the phase, which acts as matrix and the interfacial tension forces between phases, which tend to restore the droplet to a spherical shape. The aforementioned ratio is called the capillary number (*Ca*) that in a shear flow field take the following expression (Equation (1)) [31],

(1) $$Ca=\frac{\eta_{m}\dot{\gamma }R}{\Gamma_{d,m}}$$

where (*η_m_*) is the shear viscosity of the matrix and the shear rate ($\dot{\gamma }$) and, the interfacial stresses by the interfacial tension between the phases (*Γ_d,m_*) and radius of the droplet (*R*). 

Based on Taylor’s analysis for Newtonian fluids, Grace [30] studied the deformation and breakup of the dispersed phase relating the *Ca* and the shear viscosity ratio (*p*) between the dispersed phase (*d*) and the matrix (*m*) defined as (Equation (2)):

(2) $$p=\frac{\eta_{d}}{\eta_{m}}$$

**Figure 1:**
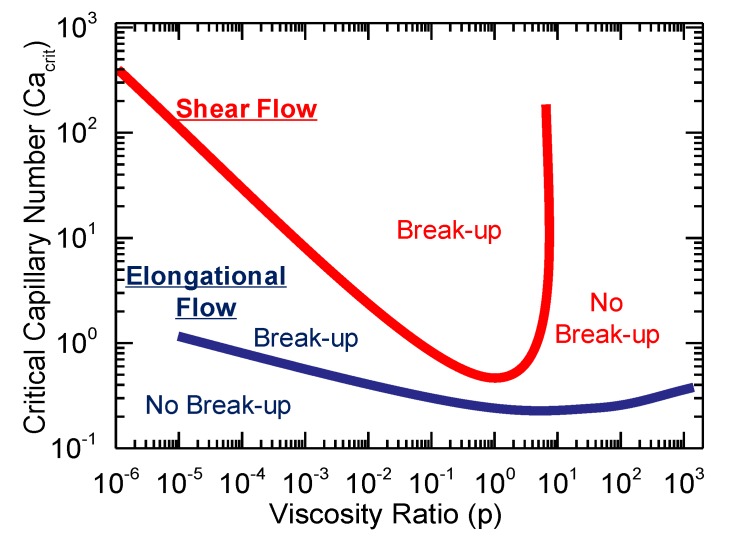
Relation between Critical Capillary Number (*Ca_crit_*) and viscosity ratio (*p*) in polymer blends according to Grace’s analysis. Adapted and modified from [30].

According to his analysis, the break-up of the initial droplet appears when a critical value of *Ca* (*Ca_crit_*) is reached, where the value depends on the viscosity ratio (*p*) and the flow field applied, which shows the trend presented in Figure 1. Furthermore, Grace observed that different deformation and break-up mechanisms can be observed depending on *p*, which are summarized in Table 1 [12].

However, it should be considered that blends of immiscible polymers are more complex fluids due to their viscoelastic nature. Therefore, the elastic component, responsible for the tendency of deformation recovery, must be taken into account since it will affect the overall morphology obtained in the blend [32,33]. Thus, depending of the type and the intensity of the stress flow field the morphology would depend as well on the elasticity ratio (*k*’) of the polymeric blend components, i.e., the one acting as matrix (*m*) and the one acting as dispersed phase (*d*). This ratio can be evaluated based on the Weissenberg number at a given shear rate ($\dot{\gamma }$) of the phases involved as follows (see Equation (3)):

(3) $$k^{\prime }=\frac{Wi_{d}\left(\omega \right)}{Wi_{m}\left(\omega \right)}$$

Yousfi et al. [16], worked on PLA/PA11 and PLA/PA6 blends, estimated *k’* from the shear oscillator rheometry measurements. Assuming that both fluids behave like Maxwell fluids and applying the Cox-Merz rule, *k’* can be determined from to the storage (*G’*) and the loss (*G”*) modulus of each blend component as a function of the angular frequency (*ω*) according to the following expression (Equation (4)):

(4) k′=G′d(ω)G″d(ω)G′m(ω)G″m(ω).

According to Van Oene [28], in order to promote the droplet/fibre transition, this ratio should be <<1, and, at same time, the viscosity ratio (*k*) should be <4. 

However, the flow field is complex in the usual processes of blend compounding such as extrusion. A set of processing conditions (temperature profile, screw rotation speed, and take-up conditions) result in the type and magnitude of the flow and, *p* and *k’* can vary point-to-point, i.e., from the screw metering zone to the exit of the die. So they can induce a number of changes in multi-phase systems, which will affect either, the drops or the whole system. The dispersed or minoritary phase can:

(i) undergo deformation from a spherical shape to ellipsoids or fibrils,
(ii) break-up into smaller ones, or
(iii) coalesce when they collide, depending on how well the morphology has been stabilized (compatibilized).

**Figure 2:**
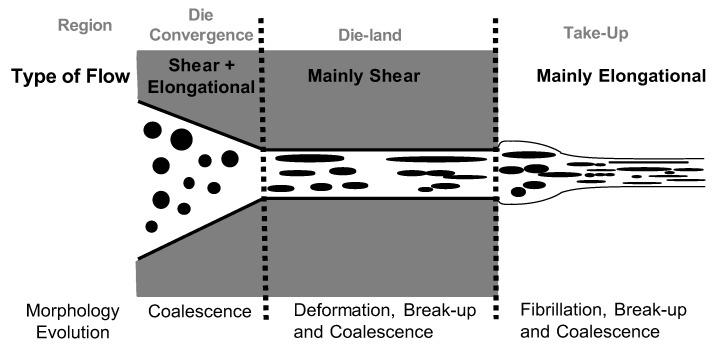
Relation between type of flow and induced morphology in different zones of an extrusion die.

The die region is extremely important. A representation of the possible morphology changes based on the flow fields applied is shown in Figure 2. The convergent flow into the die produces strong elongational forces that, on one hand, elongate the dispersed drops into fibres, which may disintegrate into fine droplets downstream from the die (die-land region). On the other hand, the convergence induces coalescence. The net effect of these two competing influences depends on the nature of the blend and how well its morphology has been stabilized. In the die-land region, the flow is mainly shear, but the mechanisms present in this zone can vary depending on the position between the walls. A droplet-to-fibril deformation mechanism is favoured near the wall where the shear field is higher. However, a coalescence mechanism is favoured at the centre [34]. 

Finally, depending on the take-up velocity, the extrudate is exposed to a hot stretching process that can considerably modify the previous morphology obtained in the die, producing a fibrillation of the dispersed phase. Li et al., working with HDPE/PET, found that the morphological characteristics of the dispersed PET phase in the blend at a fixed weight composition (15% w/w of PET), an extrusion temperature profile and screw rotation speed were dependent on the hot stretching ratio. As the hot stretching ratio was increased, the PET particles changed from spheres (HS = 1, no stretching) to ellipsoids, to rodlike particles (HS = 11), and finally to microfibrils (HS = 47) [35].

The aim of the present study was to explore the ability to control the morphology obtained in PLA/PA bio-blends, considering the rheological criteria related to shear viscosity and elasticity of the phases, and the way they can be regulated by extrusion variables, such as the screw rotation speed and take-up velocity of the extrudate. The resulting thermo-mechanical behaviour of the blends are related to the resultant morphology obtained.

## 2. Materials and Methods

### 2.1. Materials

Bio-blends were prepared using as matrix two forms of PLA. One of the PLA form is a commercial Poly(lactid acid) grade (Ingeo 4032D^®^) from NatureWorks (Arendonk, Belgium), with a D-lactide molar content of 2%, a *M*_n_ of 90,000 g/mol and *M*_w_ of 181,000 g/mol, and a melting temperature (*T*_m_) of 167 °C. 

The second type of matrix was a rheologically modified form of the previous PLA, referred to later on as PLA_REx._ This modification was obtained by a reactive extrusion (REx) process following the same procedure and conditions previously reported in [36], using as reagent a styrene-acrylic multifunctional-epoxide oligomeric agent (Joncryl-ADR-4400^®^, kindly supplied by BASF, Ludwigshafen, Germany), with an epoxy equivalent weight of 485 g/mol and a functionality of 14. Under these processing conditions, chain extension and sparsely three-arm star branching are promoted in PLA which results in a content of approximately 24% w/w of modified chains, causing an increase in its melt elasticity [18,37].

As dispersed phase, a bio-based PA10.10 (Zytel RS LC1000 BK385) was used, manufactured by DuPont (Wilmington, DE, USA), with a melting temperature (*T*_m_) of 200 °C, a *M*_n_ of 11,000 g/mol, and *M*_w_ of 33,000 g/mol. 

The blend compositions and codes used along this work are summarized in Table 2.

### 2.2. Bio-blends Preparation

**Figure 3:**
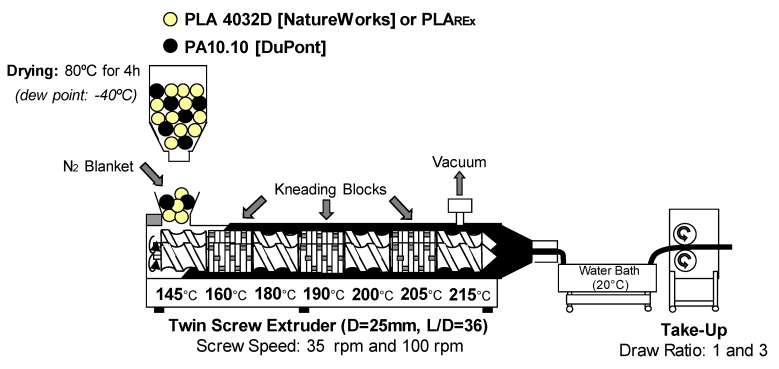
Scheme of the processing method employed for bio-blend preparation through twin-screw extrusion indicating the processing conditions used.

Bio-blends were prepared by melt mixing in an intermeshing co-rotating twin-screw extruder, featuring three kneading blocks, each 100 mm in length equally distributed along the screws length (Kneter 25X24D, COLLIN Lab and Pilot Solutions GmbH, Maintenbeth, Germany), using a circular cross section die with a nominal diameter of 3 mm and a temperature extrusion profile of 145, 160, 180, 190, 200, 205, and 215 °C from the feeding zone to die, respectively (See Figure 3). The distance between the die-exit and the first contact of the extrudate with the cooling bath (at 20 °C) remained fixed at 100 mm. 

In order to evaluate the effect of the shear rate ($\dot{\gamma }$) on the morphology, two different screw rotation speeds were selected: 30 and 100 rpm. The effect of the hot stretching process on the morphology was evaluated by applying three take-up velocities (regulated by rpm of the take-up rolls located after the cooling bath) such that nominal draw ratios (DR) of 0 (no stretch), 1, and 3 were obtained. The nominal DRs were estimated according to the following expression (Equation (5)):

(5) $$draw\;ratio=\frac{Cross\;section\;of\;the\;die\;exit\;}{Cross\;section\;of\;stretched\;extrudate}$$

A preliminary study showed that, under the used processing conditions, all extruded filaments showed no crystallinity of the PLA phase, according to the differential scanning calorimetry (DSC) results obtained from the first heating cycle (10–230 °C) at 10 °C·min^−1^.

Prior to all the extrusion compounding, the materials were always dried at 80 °C for 4 h in a Piovan hopper-dryer (dew point = −40 °C) and processed with a N_2_ blanket in the feeding zone to prevent a possible degradation. Vacuum was applied in the metering zone to remove volatiles created during the reactive stage.

### 2.3. Rheological Characterisation

The rheological characterization of the pristine polymers and prepared bio-blends was performed using a Small-Amplitude Oscillatory Shear Methodology (SAOS). An AR-G2 rheometer (TA Instruments, New Castle, DE, USA) was used under dry N_2_ atmosphere in parallel plate configuration with a constant gap of 1 mm. Prior to testing, pellets were vacuum-dried overnight at 55 °C over silica gel. Measurements were performed in the linear viscoelastic region under controlled deformation conditions at 2%, the dynamic frequency sweeps at 215 °C (i.e., the blend processing temperature) were in the frequency (*ω*) range 0.0628 < *ω* < 628 rad/s.

### 2.4. Morphological Characterization

**Figure 4:**
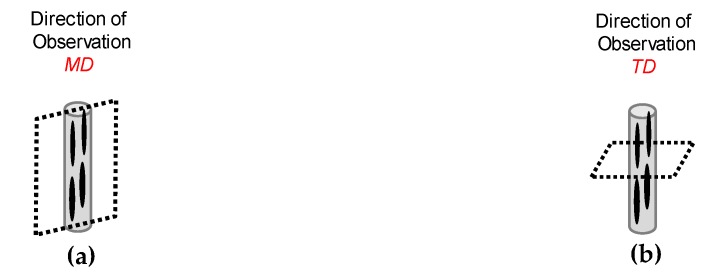
Scheme of the observation directions for the morphology analysis: (**a**) melt flow direction (MD); (**b**) transversal to melt flow direction (TD).

Bio-blend morphologies were assessed at different directions of observation, as proposed in Figure 4, using a scanning electron microscope (JSM-7001F, JEOL Ltd., Tokyo, Japan); the accelerating voltage used was 2 kV. The samples were broken under cryogenic conditions and a selective etching was performed to improve the morphology analysis. The samples were immersed in a dissolution of sodium hydroxide at 0.025 mol/L in water with methanol, at a volumetric concentration of 1:2, respectively, for 96 h at 23 °C. All the samples were coated with platinum-palladium 80–20 wt % prior to the observation.

Image analysis was made to representative zones of the SEM images with an open-source software (ImageJ version 1.51p, NIH, Bethesda, MD, USA). A discrete distribution of sizes was determined by histograms that were adjusted to a lognormal function. The number-average particle size ${\bar{d}}_{n}$ was determined based on the adjust for several regions in the bio-blends, according to Equation (6), where *n_i_* is the number of dispersed domains and *d_i_* its size. Meanwhile, the dispersion was measured with the full width half maximum of the distribution:

(6) $${\bar{d}}_{n}=\frac{\sum n_{i}d_{i}}{\sum n_{i}}.$$

### 2.5. Thermo-Mechanical Characterisation

A dynamic thermo-mechanical analyzer (DMA Q800, TA Instruments, New Castle, DE, USA) was used to obtain the thermo-mechanical properties spectrum: Storage Modulus (*E’*), Loss Modulus (*E”*), and the specific loss (*tan δ*). The filaments were tested using a tensile mode at 1 Hz and 0.02% strain with a temperature range from 30 to 106 °C at 2 °C/min. A static force of 0.01 N and a force track of 125% were used. 

## 3. Results and Discussions

The results and discussions are presented in two sections: First, a rheological study of the pristine polymers and bio-blends to evaluate the potential to produce a fibrillated morphology, and; a second part based on the analysis of the relationship between the morphology obtained, using different extrusion parameters, and the thermo-mechanical properties of the bio-blends. 

### 3.1. Microfibrillation Potential Using a Rheological Analysis

Applying Cox-Merz rule as a valid approximation for the pristine polymers, the steady-shear viscosity can be applied to correlate with complex viscosity. In this study, the maximum shear rate could be found in the die and can be calculated using Equation (7), where *r_die_* is the radius of the die and *Q_v_* the volumetric flow that will depend on the extrusion parameters and the pristine polymer used [38]. This preliminary study was based in the shear rates obtained during the preparation of the bio-blends; the values of shear rate found were around 161 to 332 s^−1^, which is equivalent to the same range in angular frequency:

(7) $${\dot{\gamma }}_{die}=\frac{4Q_{v}}{\pi {\left(r_{die}\right)}^{3}}\;.$$

**Figure 5:**
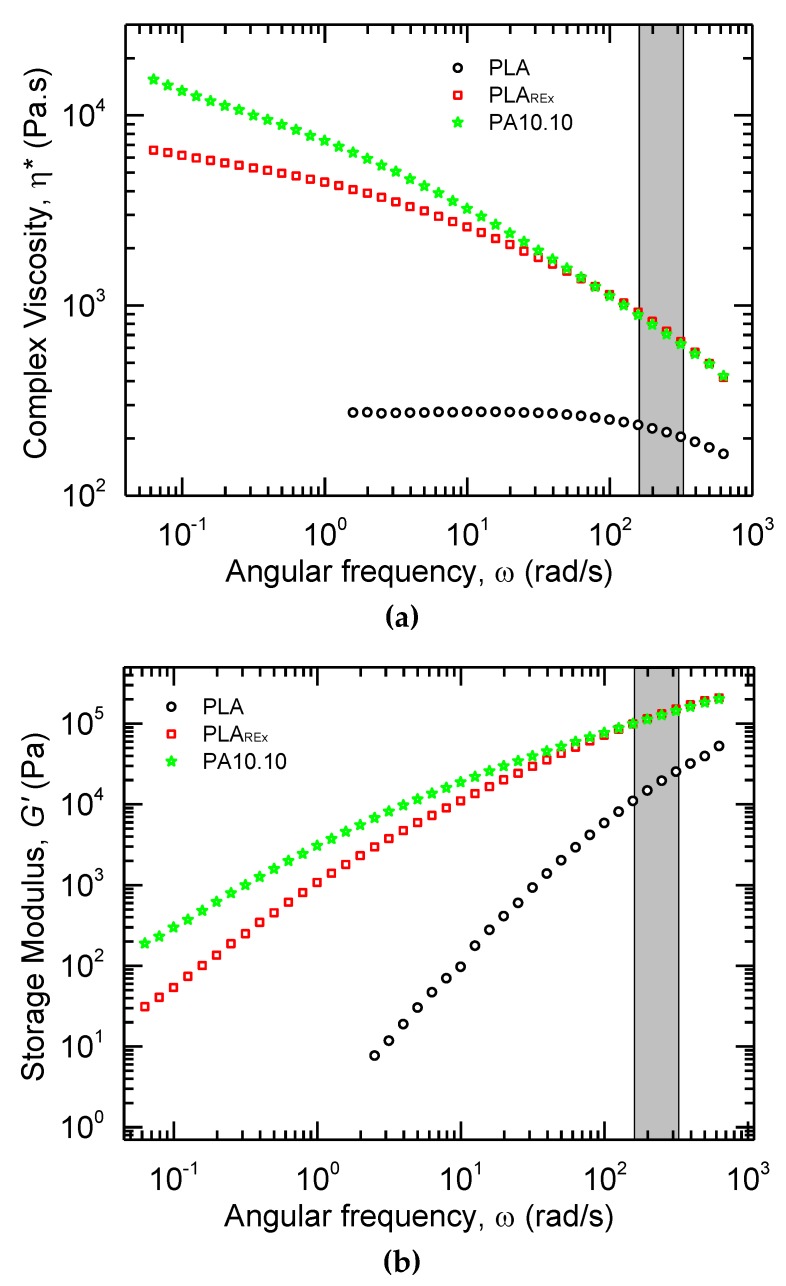
Rheological behavior in the pristine polymers at 215 °C. (**a**) Complex Viscosity (η*) versus angular frequency (*ω*), (**b**) Storage Modulus (*G’*) versus angular frequency (*ω*). The shadowed region in grey represents the equivalent angular frequencies (*ω*) used in the extrusion process to prepare the bio-blends.

The analysis of the rheological behavior of the pristine polymers is presented in Figure 5. As shown in Figure 5a, the neat PLA presented a Newtonian behavior in frequencies lower than ten (10) rad/s. In contrast, the PLA_REx_ showed a shear-thinning region with a significant increase in viscosity compared to the neat PLA and did not reach the Newtonian region. In the area of interest for the extrusion process, the viscosity increase was over a magnitude scale of difference. These results were expected due to the structural modification by the chain extension and the branching of the reactive extrusion, which increase the number of interactions and entanglements per chain, altering the molecular mobility [39,40]. Moreover, the PA had a similar behavior to the PLA_REx_ with a complete clear shear thinning region in the range tested and higher strain rate sensitivity with the shear rate. Also, PA was presented upward in the trace, which can be associated with a post-condensation process in low frequencies, and has been found in studies of PA11 and PA6 [16,41,42].

In relation to the storage modulus (*G’*) presented in Figure 5b, the PLA had the lowest values and reached the terminal region. This represented a lower elastic component that could produce a less effective transmission of stress in the secondary phase during mixing. In the case of the PLA_REx_, the *G’* increased considerably and did not reach the terminal region. This can be explained by the possible increase of entanglements and change in the mobility of the chains [39]. 

**Figure 6:**
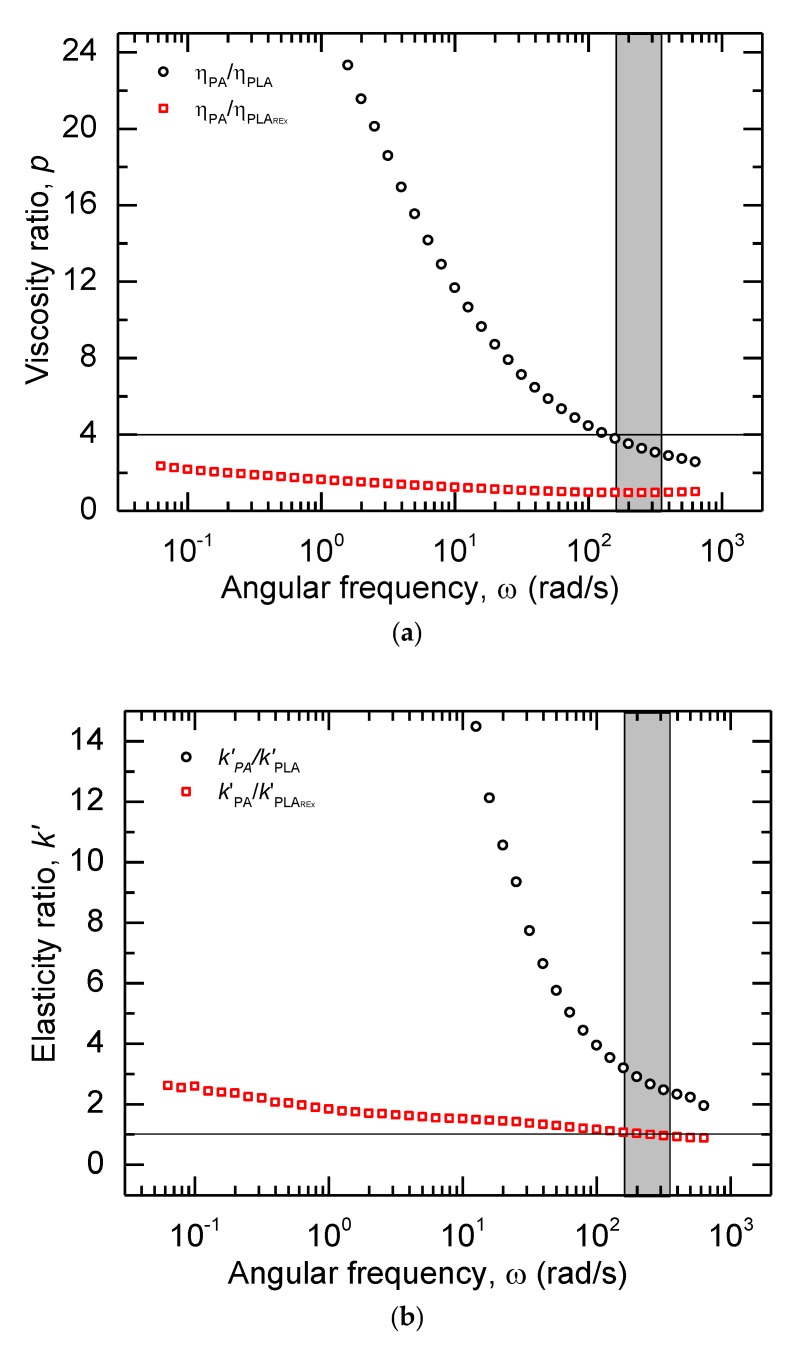
Relative Rheological Parameters between the pristine polymers versus the angular frequency (*ω*) at 215 °C: (**a**) Viscosity Ratio (*p*); (**b**) elasticity ratio (*k’*). The shadowed region in gray represents the equivalent angular frequencies (*ω*) used in the extrusion process to prepare the bio-blends. The horizontal line marked in the graph represents the limit values of viscosity ratio (**a**) and elasticity ratio (**b**) to produce a fibrillated morphology.

The viscosity ratio (*p*) between the two phases evidenced a tendency to decrease due to the difference in strain rate sensitivities of each material (See Figure 6). When using PLA as matrix, the change in the *p* was more pronounced due to the significant difference between the viscosities, the PA with a shear-thinning behavior over the whole angular frequency range, and the PLA with a Newtonian behavior. On the other hand, using PLA_REx_ as matrix, the *p* in the complete range of extrusion is near the unit due to the similarities in the PA and the PLA_REx_. The viscosity ratios, in both kinds of materials for the extrusion shear rate, could produce a behavior that promotes a break-up in the secondary phase when the ratio falls below 4, as proposed in Table 1 [16,28].

Furthermore, the elasticity ratio (*k’*) between the pristine polymers of the bio-blends obtained had a different behavior, depending on the kind of PLA used. For the PLA matrix, an extensive scope of *k’* in the extrusion range was found, which produced values higher than 2 when the range to produce the maximum elongational deformation of the droplets had estimated values around 1. This matrix failed the theoretical elasticity ratio requirements. However, the PLA_REx_ with the similarity in the rheological behavior of the pristine polymers produced a *k’* near 1, which could lead to the formation of a fibrillated morphology in the second phase, as predicted by Mighri et al. and used by Yousfi et al. [16,28,43,44].

**Figure 7:**
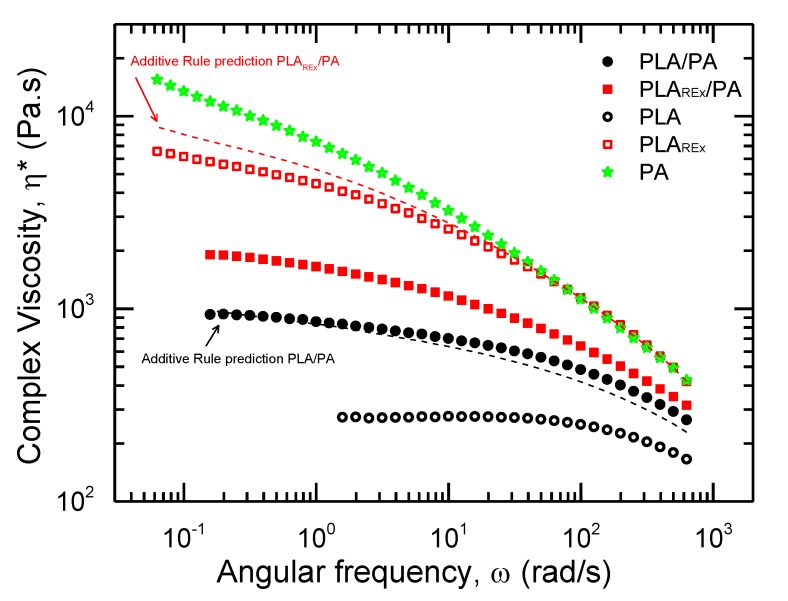
Complex viscosity (η*) vs. angular frequency (*ω*) at 215 °C of pristine polymers (hole scatter points) and prepared bio-blends (filled scatter points). Dashed lines represent the prediction of the additive rule.

A SAOS study was performed in order to study and elucidate any change in the rheological behavior in the bio-blends prepared and to get more insight. Although, the values of viscosity increased as compared to the viscosity of neat PLA, as shown in Figure 7, the PLA bio-blend had a shear-thinning behavior in almost all the frequency range studied. It did not completely follow an additive rule of mixture (ARM) behavior between pristine polymers, showing a positive deviation in frequencies higher than 1 rad/s [40,45].

The PLA_REx_ bio-blend showed a lower viscosity in all *ω* compared to the pristine polymers. The behavior obtained had a negative deviation compared to the additive rule prediction. This anomaly behavior has been associated with an interfacial slip between the phases in the interface. The interface between the phases during imposed stress can produce drainage due to a decrease in the viscosity of the zone between two PA domains. This phenomenon can occur when the entanglements of the dispersed phase in the interfacial zone are lower than the bulk material [26,45,46,47]. 

**Figure 8:**
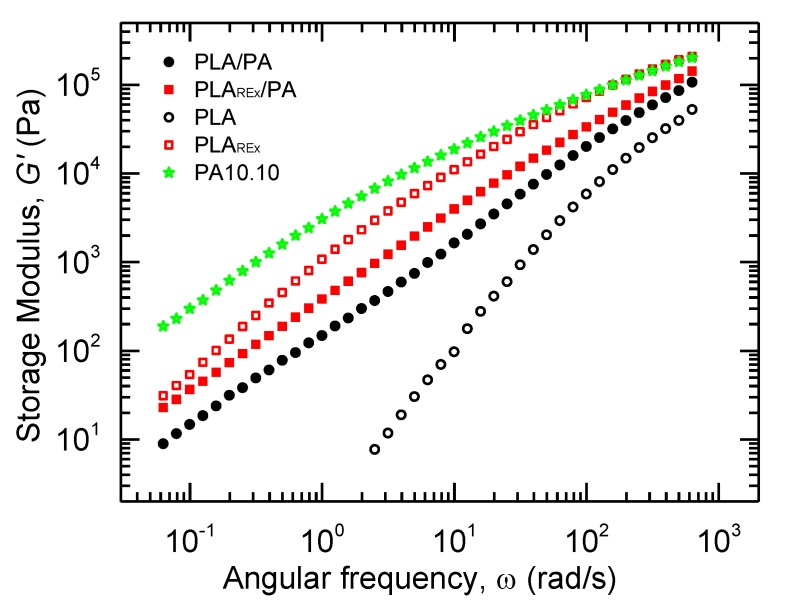
Storage modulus (*G’*) versus angular frequency (*ω*) at 215 °C of pristine polymers (hole scatter points) and prepared bio-blends (filled scatter points).

*G’* of the bio-blends are shown in Figure 8. The PLA_REx_ bio-blends presented a higher elastic behavior as compared to the PLA bio-blends. This can be related to the higher elastic behavior of PLA_REx_ as compared to PLA. It is worth noting that PLA bio-blends showed a shoulder around 4 rad/s in *G’*. This could reveal the existence of two well defined phases, one of them dispersed with different elasticity in the system due to a biphasic nature produced by the changes in the relaxation times [48]. Furthermore, the PLA_REx_ bio-blends had a smooth shoulder for lower frequencies. According to Palierne, this could be attributed to a decrease in the sizes of the second phase, or the diminishing of the interfacial tension between the phases [25,31]. 

**Figure 9:**
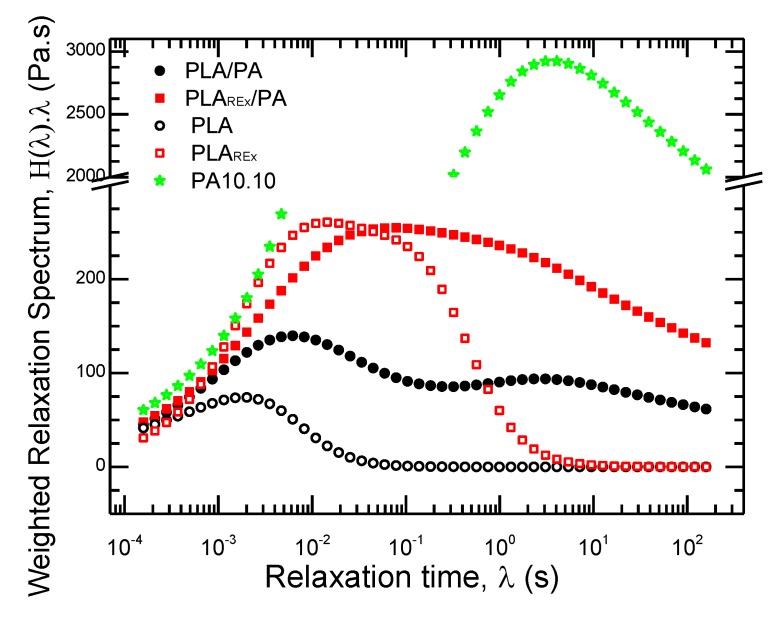
The weighted relaxation spectrum at 215 °C of pristine polymers (hole scatter points) and prepared bio-blends (filled scatter points).

The weighted relaxation spectrum was calculated using the commercial Rheology Trios software (TA Instruments, New Castle, DE, USA). As observed in the spectrums in Figure 9, the PLA_REx_ produced a widening and an increase in the maximum relaxation time from 10^−3^ to 10^−2^ s as compared to neat PLA. This could be attributed to a change in the molecular weight due to the ramifications generated by the addition of the chain extender which produces a system with a more restricted mobility [39]. The PA presented a unimodal distribution with a wide amplitude centered at 4s. 

Furthermore, the PLA bio-blends had a bimodal distribution with a maximum time of relaxation centered at 1 × 10^−2^ s which corresponds to the PLA phase. This evidences an increase from the pristine polymer value of 2 × 10^−3^ s. Also, the peak for the PA phase appeared to maintain a time similar to the pristine polymer, near 3 s [25]. Both relaxation times could be related to the presence of two defined phases without interaction in the molecular mobility between them. 

On other hand, the PLA_REx_ bio-blends appeared to have an overlapped bimodal distribution with a shift in the maximum times of relaxation as compared to those of the pristine polymers. The maximum time related with the PLA_REx_ phase increased to 3 × 10^−2^ s and the one related to the PA phase decreased to 1 s. These results indicate that there was more interaction between the phases due to the use of the chain extender that could decrease the interface tension, as proven by Yousfi et al. in blends of PA11. 

**Figure 10:**
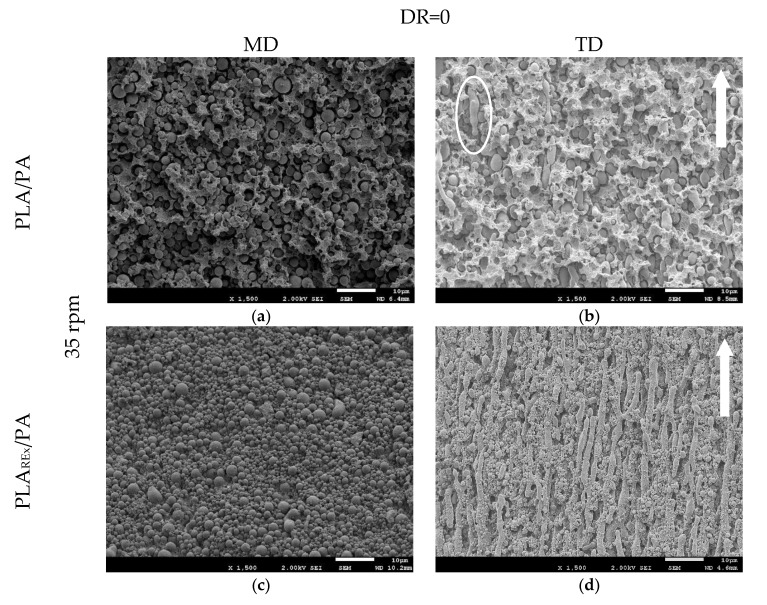
SEM Micrographs of bio-blends at a screw speed rotation of 35 rpm. (**a**) and (**b**) PLA/PA bio-blend TD and MD observation, respectively; (**c**) and (**d**) PLA_REx_/PA bio-blend TD, and MD observation, respectively.

Figure 10 shows the SEM micrographs in the TD (Transverse to flow direction) and MD (Parallel to flow direction) observation planes for unstretched extruded blends at a screw rotation speed of 35 rpm. Analyzing the TD direction, a sea-island structure was found for both bio-blends and it can be clearly seen that the use of PLA_REx_ as matrix of the blend (Figure 10c) favoured a better dispersion of the secondary (minority) phase, showing a decrease in particle size as well as a narrower size distribution. The observation was corroborated with the quantitative analysis performed (Table 3). This situation would be expected as *p* is lower than 1 for these blends, which would promote the mechanisms of “End Pinching” and “Necking break-up” referenced in Table 1. On the other hand, it would lead to a lower probability of coalescence that could occur in the convergent flow zone in the head (see Figure 2), as a consequence of the increase in “surface energy” that the reactive modification causes in the PLA_REx_ and that has been reported by Cailloux et al. [18]. According to these authors, the surface energy of PLA is 46.4 mJ/m^2^ vs. 51.5 mJ/m^2^ for PLA_REx_.

However, when analyzing the MD views, as shown in Figure 10b,d, it can be seen that the PLA_REx_ bio-blends actually presented a fibrillar morphology in the extrusion conditions used. This was expected considering the relationship of elasticity between the phases that occurs in this case (next to 1). This aspect shows that the elasticity of the PLA_REx_ phase was great enough to transmit the stresses generated during the flow (combination of shear and elongation in the die-land) and deformed the drop of PA to generate the elongated morphology, in apparent contradiction with the shear viscosity ratio (*p*) criteria (See Table 1). In the case of PLA bio-blends, the lower elasticity of the matrix is thought to make the transmission of stress between phases less effective. Even considering the greater elasticity of PA, the chances of reaching the conditions for the phenomenon of Rayleigh instability as a precursor to the elongated gout rupture are greater. Evidence of this process can be seen in several regions of Figure 10b. To this aspect, it should be added that the probabilities of dispersed phase coalescence are slightly higher in this system, considering the lower surface energy that PLA presents in this temperature range [28,32].

Similar trends have been observed for the bio-blends processed with a screw rotation speed of 100 rpm.

### 3.2. Morphological Analysis of the Hot Stretched Bio-blends

**Figure 11:**
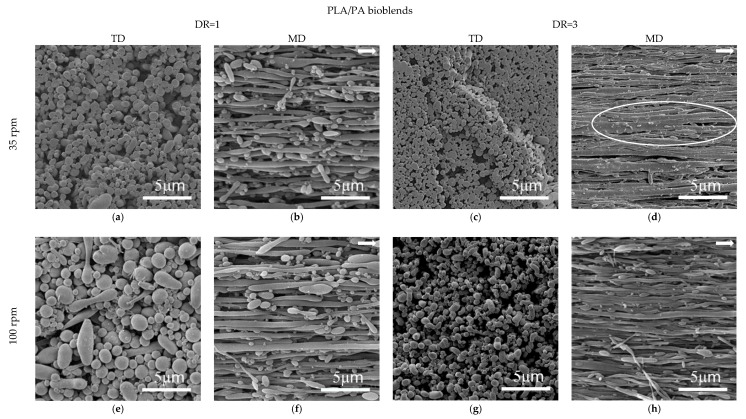
SEM micrographs of hot stretched PLA/PA bio-blends at the conditions referenced in the figure. Arrows represent the melt flow direction.

**Figure 12:**
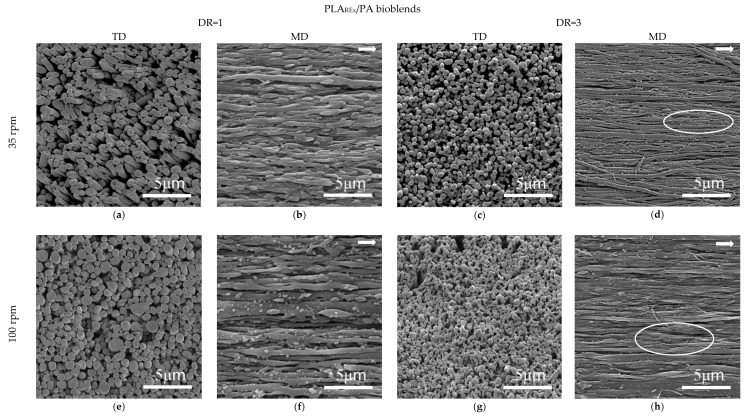
SEM micrographs of hot stretched PLA_REx_/PA bio-blends at the conditions referenced in the figure. Arrows represent the melt flow direction.

**Figure 13:**
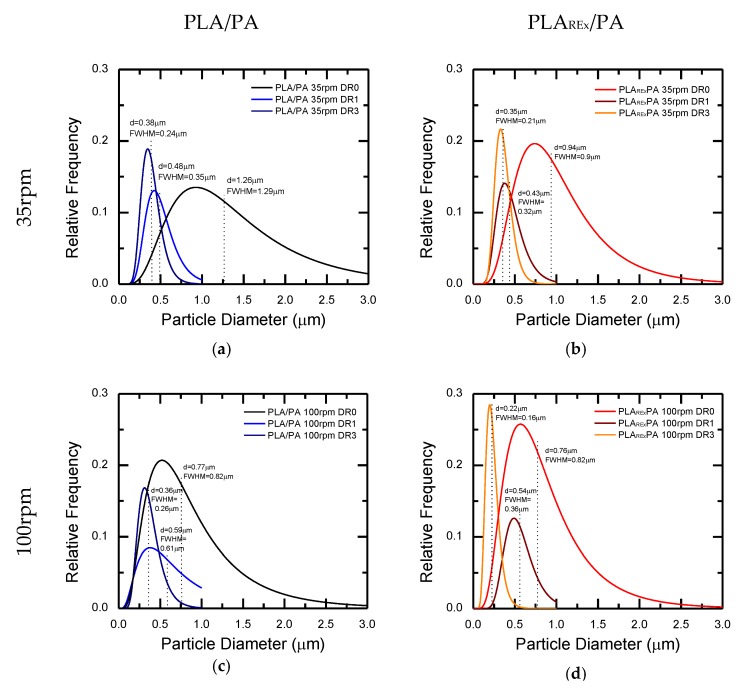
Size distribution of the PA domains determined by image analysis from SEM micrographs in TD observation as function of the extrusion screw rotation speed (rpm) and draw ratios (DR) in the hot stretching stage. (**a**) and (**b**) PLA/PA and PLA_REx_/PA at 35rpm, respectively; (**c**) and (**d**) PLA/PA and PLA_REx_/PA at 100 rpm, respectively.

Figure 11 and Figure 12 show the SEM micrographs in the TD and MD observation planes for the different processing conditions, with stretching applied at the die exit of the blends, based on PLA (PLA/PA), and PLA_REx_ (PLA_REx_/PA), respectively. Figure 13 shows the distribution of sizes obtained in these, after the quantitative analysis performed.

Generally, the combined view of both observation planes shows the generation of a fibrillar morphology with different degrees of slenderness (aspect ratio and localized coalescence, depending on the matrix and levels of stretching applied). Focusing on the PLA/PA bio-blends, the aspect ratio of the fibrils that are generated at low DR (DR=1) decreased with increasing screw rotation speed (see micrographs a,b,e, and f in Figure 11). Possibly, for this extrusion condition, the probability of coalescence of the PA phase within the converging region of the die increased, generating “precursor” droplets of greater diameter. In this case the fibrils will have a larger diameter for the same magnitude of extensional stresses generated in the die exit by the take-up speed applied. Under these conditions it appeared that the chances of reaching Rayleigh instability were higher, given the high amount of oblong structures that appear in the MD observations. A similar but less obvious trend is seen for PLA_REx_-based bio-blends coupled with some coalescence of fibrils for a screw rotation speed of 100 rpm (see Figure 12d).

Increasing the DR to 3 resulted in a general increase for all blends in the aspect ratio of the fibrils generated, along with the narrowing in the distribution of their diameters (see Figure 13) which is a much more marked effect for PLA_REx_-based bio-blends. It is noteworthy that in this case (DR = 3), regardless of the screw rotation speed used, a certain degree of interconnection between fibrils can be seen (see encircled regions in Figure 11 and Figure 12). This interconnection can be a consequence of the squeezing effect between two adjacent PA filaments on the PLA or PLA_REx_ phase, which promotes drainage and subsequent local coalescence between filaments and which is favored when low interfacial tension between phases is observed [49].

### 3.3. Dynamic Mechanical-Thermal Analysis 

**Figure 14:**
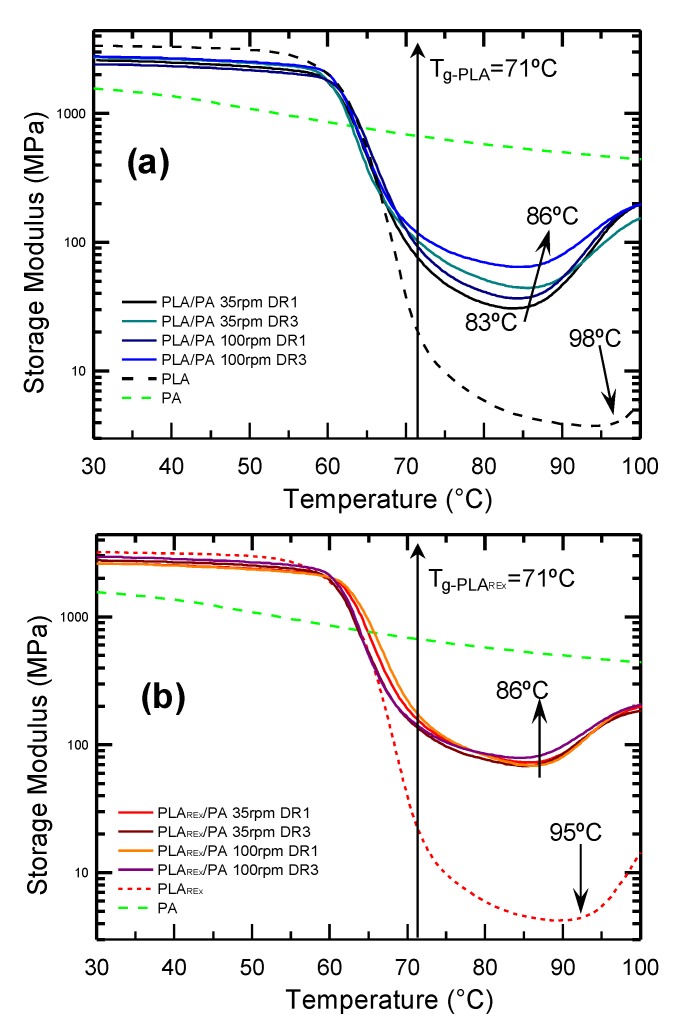
Storage Modulus (*E’*) variation with temperature for the different processing conditions (extrusion screw rotation speed, rpm, and draw ratios, DR): (**a**) PLA/PA and (**b**) PLA_REx_/PA bio-blends and their respective pristine polymers.

Figure 14 shows the storage modulus (*E’*) spectra obtained for the studied materials. As can be seen, the most appreciable differences occurred in the rubbery region. The simple addition of PA promoted a decrease of around 10 °C, whereas the effect related to cold crystallization of the system is presented, which in the case of PLA is located at 98 °C and for PLA_REx_ at 95 °C. The greatest decrease occurred in those systems where the aspect ratio (slenderness) of the PA fibril was high (mixtures with higher DR applied). A similar trend has been observed by Park et al. [14] in PLA/PA6 mixtures, which they attributed to a nucleating effect of the PA phase. This becomes more pronounced by promoting microfibrillation due to the increase in the specific surface area by providing more heterogeneous nucleation sites to the crystals. 

Another interesting detail is that the addition of PA in the form of microfibrils caused a notable increase in *E’* throughout this region, between 500% and 1300% with respect to the PLA in the PLA/PA bio-blends, and between 1300% and 1600% for the PLA_REx_/PA bio-blends.

Both observations are especially relevant where the target of the mixtures under study is considered: FDM processing, especially at the deposition stage. This improvement provides greater mechanical stability of the filament deposited in the substrate, which would gain accuracy in the process. Additionally, the maximum service temperature range of the finished product could increase, given cold crystallization at lower temperatures may generate an increase in the crystallinity of the previously deposited layers. This is due to an annealing effect that can be generated when the second layer is deposited.

**Figure 15:**
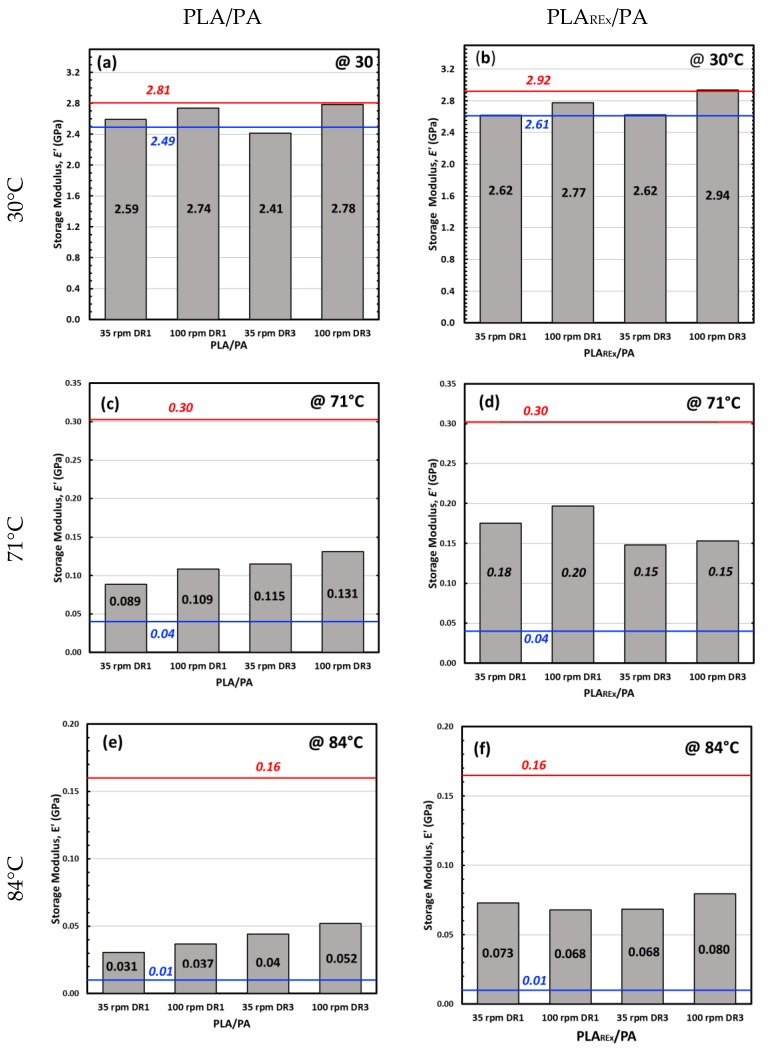
Storage moduli (E’) determined at: (**a**), (**b**) 30 °C for PLA/PA and PLA_REx_/PA bio-blends, respectively; (**c**), (**d**) at 71 °C for PLA/PA and PLA_REx_/PA bio-blends, respectively; (**e**) and (**f**) at 84 °C for PLA/PA, and PLA_REx_/PA bio-blends, respectively. Red line represents the upper limit model. Blue line represents the lower limit model.

A detailed analysis of the obtained parameter values is presented in Figure 15. In this case the *E’* has been considered in three characteristic regions of the spectrum: in the glassy region (30 °C), at the *Tg* value of each respective matrix without blending (PLA and PLA_REx_) (taken as the temperature at the maximum value of specific loss (*Tan δ*), around 70.6 °C), at 84.5 °C, the mean temperature, where the bio-blends showed the onset of the new increase on *E’* in the rubbery region associated to the effect of cold crystallization. It should be taken into account that, according to a preliminary DSC study, the matrix in both groups of blends did not show evidence of crystallinity in any of the processed blends. With respect to the PA phase, the crystallinity ratio did not show any variation with the processing conditions.

In the same figure, predictions made by the additive model for long fibre composites (i.e., with a fibre length greater than the critical reinforcement length) with good adhesion to the matrix have been included. This model considers that the maximum value that the composite can reach (upper limit) is when both fibril and matrix deform equally during mechanical solicitation (Iso-strain condition), while the lower limit of prediction occurs when both phases bear the same tension; but, as a consequence of a moderate adhesion and/or disposition of the dispersed phase, each one is deformed independently.

In the glassy region of the material (30 °C) (Figure 15a,b), both bio-blends, with the exception of PLA/PA, processed at 35 rpm with a DR = 3 presented values within the prediction window. In this case it should be recalled that this blend was the one with a relatively high degree of interconnection or coalescence (Figure 11c,d). This causes the specific surface area to decrease, limiting its potential reinforcement capacity and may not even reach the critical fibre length dimensions to act as an effective reinforcement. It is important to highlight that in both types of bio-blends, those prepared at 100 rpm of screw rotation speed and with an applied DR of 3, reach the value predicted by the upper limit of the long fibre composites model. This indicates that the slenderness reached by the PA fibrils exceeds the critical length so that they act as long fibre reinforcement.

At the *Tg* of the respective unmixed matrices, both bio-blends enter into the model prediction window, being closer to the lower limit in the case of the bio-blend PLA/PA (Figure 15c). It is noteworthy that *E’* increased as the DR increased for this blend, which did not occur for PLA_REx_ based bio-blends (Figure 15d). However, considering the magnitude of values that were measured (88 to 197 MPa), any trend must be considered with caution. What is clear is that PLA_REx_-based blends have higher values in this temperature range.

At the onset of the cold crystallization process of the blends (around 84–85 °C), the trend is similar to that observed in the temperature range discussed previously. In this case, for the PLA bio-blends, an increase in DR promoted an increase in *E’* (Figure 15e), coinciding with the generation of highly microfibrillated morphology presented (Figure 11g,h). On the other hand, the PLA_REx_ bio-blend had virtually no dependence on the extrusion parameters used, showing in all cases values above those obtained for the PLA bio-blends.

It is important to note that caution must be taken when trying to correlate the morphology obtained and evaluated at room temperature (Figure 11 and Figure 12) with the mechanical parameters evaluated above the *Tg* of the matrix. As a consequence of the greater molecular mobility at these temperatures, spinodal decomposition may occur, promoting phase coalescence. This phenomenon can be much more pronounced in mixtures based on PLA if one considers the lowest interfacial tension that these mixtures present, especially in those with morphology in which the degree of fibrillation is not that high (Figure 11a,b,e,f).

## 4. Conclusions

SEM observations revealed that, unlike neat PLA, PLA**_REx_** promoted PA-**μ**f without hot stretching (DR = 0). This observation agrees with the predicted viscosity (***p***) and elasticity (***k’***) predictions at the temperature and shear rates estimated at the die. For neat PLA based blends PA microfibrillation could be promoted, thereby inducing a hot stretching stage during the take-up process.

As the DR increased, the general effect for all blends was that the aspect ratio of the fibrils generated increased along with the narrowing in the distribution of their diameters, being much more pronounced for PLA**_REx_** based bio-blends. However, regardless of the screw rotation speed used, a certain degree of interconnection between fibrils is observed, generating a stratified-like morphology. This effect could be attributed to the low interfacial tension between phases, which promotes the PLA phase drainage due to the squeezing effect exerted by two adjacent PA fibrils. 

DMTA analysis showed that at 30 °C the storage modulus (*E’*) was close to the iso-strain predictions (upper limit) of the additive model for long fiber composites, indicating that the critical length of the fibril for effective reinforcement was reached. In the rubbery region of the blends (*T* > *Tg*_PLA-phase_), the use of PLA_REx_ promoted a better mechanical performance in the rubbery region due to the microfibrillar morphology obtained.

## Figures and Tables

**Table 1:** Break-up mechanisms in shear flow related to viscosity ratio (*p*) after the *Ca_crit_* is reached.

Viscosity Ratio (*p*)	Break-up Mechanism	Description
Much lower 0.1	Tip streaming 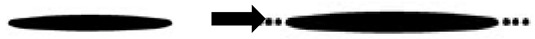	The break-up occurs at the tip of the deformed droplet and generally produce very fine droplets
0.1 < *p* < 1	Necking 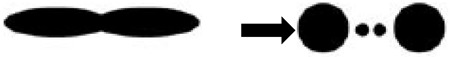	The deformed droplet breaks up into two daughter droplets.
	End pinching 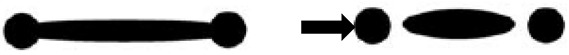	The deformation produces a dumbbell shape, after which the break-up occurs in the two droplets formed at the ends.
1 < *p* < 3.8	Rayleigh break-up 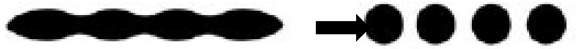	Interfacial instabilities produce disturbances that will break up the fibril into a line of droplets.
*p* > 3.8	No break-up 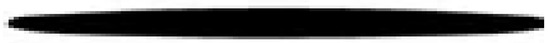	The break-up of the droplet under shear is not possible.

**Table 2:** Composition and nomenclature used in the investigated bio-blends.

Sample Nomenclature	% w/w of Pristine Polymers		
	PLA	PLA_REx_	PA10.10
PLA/PA	70	-	30
PLA_REx_/PA	-	70	30

**Table 3:** Average size and size distribution obtained from the quantitative analysis of SEM observations in TD for bio-blends processed at a screw rotation speed of 35 rpm.

	PLA/PA	PLA_REx_/PA
Mean Size (μm)	1.26	0.94
Full Width at Half Maximum (μm)	1.29	0.90
